# Educational Intervention Improves Antimicrobial Stewardship Knowledge but Reveals Persistent Barriers in Rural Hospitals

**DOI:** 10.3390/antibiotics15090859

**Published:** 2026-09-03

**Authors:** Jack H. Lambert, Rayven Todd, Thomas W. Bagwell, Damani Andre, Raybun Spelts, Fantasia Gorham, Jamie Woods, Ayomide H. Adeyemi, Shondia Evans, Rafael Ponce-Terashima, Kenneth I. Onyedibe

**Affiliations:** 1 Department of Biomedical Sciences, Mercer University School of Medicine, Macon, GA 31207, USA; jack.harris.lambert@live.mercer.edu (J.H.L.); thomas.walter.bagwell@live.mercer.edu (T.W.B.); 2 Department of Microbiology, Biochemistry & Immunology, Morehouse School of Medicine, Atlanta, GA 30310, USA; raytodd@msm.edu (R.T.); dandre@msm.edu (D.A.); ay.adeyemi05@gmail.com (A.H.A.); 3 Archbold Memorial Regional Medical Centre, Thomasville, GA 31792, USA; raybun2012@gmail.com; 4 Georgia Department of Public Health, Atlanta, GA 30334, USA; fantasia.gorham2@dph.ga.gov (F.G.); jamie.woods@dph.ga.gov (J.W.); 5 Georgia Hospital Association, Atlanta, GA 30339, USA; sevans@gha.org; 6 Division of Infectious Diseases, Mercer University School of Medicine, Macon, GA 31201, USA; ponce_ra@mercer.edu

**Keywords:** critical access hospital, rural health, antimicrobial stewardship, antibiogram, education, implementation barriers

## Abstract

**Background/Objectives**: Antimicrobial resistance threatens public health globally, and critical access hospitals (CAHs) serving rural communities face structural barriers to implementing antibiotic stewardship programs (ASPs). This study evaluated whether a regional educational intervention could improve antimicrobial stewardship knowledge and implementation among rural healthcare professionals. **Methods**: A quantitative, quasi-experimental repeated cross-sectional evaluation was conducted following Antibiotic Stewardship conferences in 2023 and 2024. Participants from 38 rural and critical access hospitals completed pre-conference, post-conference, 6-month follow-up, and implementation surveys. Quantitative data were analyzed using descriptive statistics and unpaired chi-square tests, with Fisher’s exact test substituted where expected cell counts were below 5, with significance set at *p* < 0.05. **Results**: Seventy healthcare professionals participated across both years. Correct identification of the use of an antibiogram to guide empiric (rather than definitive) therapy improved from 27% pre-conference to 58% post-conference (*p* = 0.029). Confidence in antibiogram interpretation increased from 67% to 100% post-intervention (*p* = 0.004, Fisher’s exact) but declined to 50% (5 of 10) at 6-month follow-up. Knowledge of the minimum isolate threshold required to construct an antibiogram improved from 23% (7 of 30) pre-conference to 64% (7 of 11) at 6-month follow-up (*p* = 0.026, Fisher’s exact). Despite this, prescribing intent for conditions where antibiotics are typically unnecessary showed minimal improvement: recommendations for antibiotic use in middle ear infections decreased from 50% to 36.84%, and for mpox from 20% to 15.79%. All participants reported intent to modify clinical practice; however, time constraints, technology limitations, and lack of resources were consistently cited as primary barriers for implementation across both survey years. **Conclusions**: A regional educational intervention improved short-term antimicrobial stewardship knowledge and confidence among rural healthcare professionals, but knowledge retention declined over time and structural barriers persisted. Education alone is insufficient; sustained reinforcement and rural-adapted ASP models incorporating protected ASP time, decision-support tools and external partnerships are needed to translate knowledge gains into consistent stewardship practice.

## 1. Introduction

Antimicrobial resistance (AMR) poses a critical threat to public health systems globally, leading to increased morbidity, mortality, and healthcare costs [1,2]. The overuse and misuse of antibiotics accelerate the development of multidrug-resistant organisms, undermining decades of medical progress [3]. The Centers for Disease Control and Prevention (CDC) estimates that in the United States alone, millions of antibiotic-resistant infections occur annually, resulting in tens of thousands of deaths.

Critical access hospitals (CAHs), which serve rural and underserved communities, are especially vulnerable due to structural constraints. These facilities often lack the infrastructure, personnel, and continuing education resources required to support formal Antibiotic Stewardship Programs (ASPs) [4]. Despite national guidelines advocating for the implementation of ASPs across all healthcare settings, the quality of implementation varies, and CAHs have limited resources. Georgia’s rural hospitals, many of which qualify as CAHs, face challenges such as inadequate diagnostic capacity, limited IT infrastructure, and reduced access to infectious disease specialists [4].

Previous literature supports the use of educational interventions to improve healthcare provider knowledge, behaviors, and confidence in antimicrobial prescribing [5]. Antibiograms, which are tools summarizing local bacterial susceptibilities, are a cornerstone of ASP implementation, yet their utility is limited if healthcare providers lack the knowledge or confidence to use them effectively [6,7]. This preliminary study builds on that evidence by evaluating a series of educational conferences delivered to rural healthcare professionals in Georgia in 2023 and 2024. The objectives of this study were to: (1) assess knowledge and confidence gains following the conferences; and (2) identify barriers to ASP implementation which can inform the development of sustainable solutions unique to CAHs.

## 2. Results

### 2.1. Participant Demographics

Across the 2023 and 2024 ASP conferences, 70 healthcare professionals participated. Thirty participants completed the 2023 pre-intervention survey, 19 completed the 2023 post-intervention survey, and 11 completed a 6-month follow-up survey using similar prompts to assess knowledge retention. In 2023 and 2024, 25 and 40 participants, respectively, completed post-conference implementation surveys. Participants represented 38 rural or critical access hospitals across Georgia. In 2023, pharmacists accounted for approximately 47% of respondents, followed by nurses at 43% and infection prevention specialists at 33%.

### 2.2. Knowledge and Confidence Related to Antibiotic Use and Antibiograms

**Figure 1:**
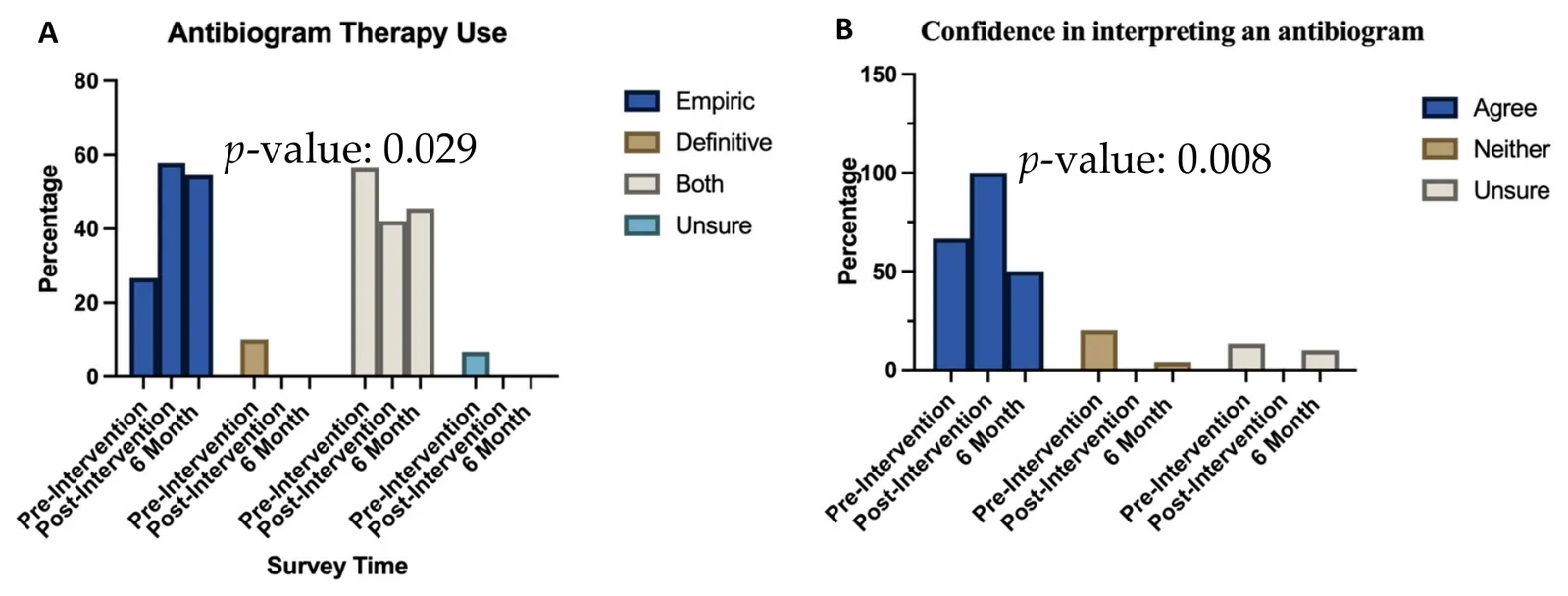
Knowledge and confidence outcomes associated with a regional antimicrobial stewardship education intervention. (**A**) Correct identification of empiric therapy as the antibiogram-guided approach (pre vs. post; *p* = 0.029). (**B**) Confidence in interpreting antibiograms at baseline, immediately post-intervention, and 6 months after (pre vs. post vs. 6-month; *p* = 0.008).

Baseline responses showed variable understanding of how facility antibiograms guide clinical decision-making. Following the intervention, correct identification of the use of a facility antibiogram to guide empiric (rather than definitive) therapy increased from 27% pre-conference (8 of 30) to 58% post-conference (11 of 19) (*p* = 0.029). Here, empiric use refers to consulting the facility antibiogram to inform initial therapy before organism-specific susceptibility results are available, as distinct from definitive, culture-directed therapy. (Figure 1A). Confidence in antibiogram interpretation increased from 67% (20 of 30) pre-intervention to 100% (19 of 19) post-intervention (*p* = 0.004, Fisher’s exact test) but appeared to decrease to 50% (5 of 10) at 6-month follow-up, a preliminary finding given the small subset of respondents at this timepoint (Figure 1B). Knowledge of the minimum isolate threshold required to construct a facility antibiogram was assessed at baseline and at 6-month follow-up. Correct responses (recognizing that 19 isolates are insufficient) rose from 23% (7 of 30) pre-conference to 64% (7 of 11) at 6-month follow-up (*p* = 0.026, Fisher’s exact test). This item was not administered in the immediate post-conference survey, so no pre-to-immediate-post comparison is available. For case-based questions, affirmative responses indicating that knowledge of common pathogens aids empiric antibiotic selection increased from 83% (25 of 30) to 95% (18 of 19), while agreement that cumulative local susceptibility data should guide treatment increased from 90% (27 of 30) to 100% (19 of 19). Despite strong understanding of antibiogram use for empiric therapy, prescribing behavior for conditions where antibiotics may be unnecessary showed minimal improvement. For instance, recommendations for antibiotic use in all middle ear infections decreased from 50% (15 of 30) to 36.84% (7 of 19) post-intervention, and for mpox from 20% (6 of 30) to 15.79% (3 of 19).

### 2.3. Planned Practice Changes and Perceived Barriers to Antimicrobial Stewardship Implementation

All participants (100%) reported a high level of intent to modify clinical practice following the intervention. The distribution of planned changes is shown in Table 1. Time constraints and technology limitations were the most frequently reported barriers to implementing these changes across both survey years (Table 1; all chi-square *p* > 0.05).

## 3. Discussion

This two-year quasi-experimental evaluation demonstrates that targeted, low-cost educational interventions can improve foundational antimicrobial stewardship program knowledge and short-term confidence in antibiogram use among healthcare professionals in rural critical access hospitals [5,8]. Improvements in understanding use of antibiograms to guide empiric therapy and antibiogram construction indicate that the intervention effectively addressed core antibiotic stewardship knowledge gaps, consistent with prior studies in resource-limited settings [6,7,8,9,10].

Despite substantial immediate gains, confidence in antibiogram interpretation declined markedly at six months, highlighting limited knowledge retention. This decline should be interpreted cautiously given the small number of respondents at follow-up (*n* = 11), but it is consistent with well-documented decay of knowledge and confidence after single-exposure educational events [6]. Contributing factors may include infrequent application of antibiogram interpretation in low-volume rural practice, the absence of reinforcement between conferences, and turnover among stewardship personnel. This pattern aligns with existing literature showing that educational interventions require ongoing reinforcement to sustain impact [10]. Longitudinal training and integration of stewardship education into routine clinical workflows are therefore needed. Practical reinforcement strategies, such as periodic booster sessions, short refresher modules, tele-stewardship strategies, including electronic medical record prompts and decision-support tools, may help reinforce knowledge and support clinical decision-making and sustain gains over time [4,9].

High reported intent to change practice indicates strong acceptability of the intervention across professional roles; however, persistent structural barriers including time constraints, technology limitations, and limited resources remain a challenge for implementation. These barriers mirror challenges previously described in rural hospitals and limited resource settings [11]. The persistence of such barriers underscores the distinction between implementation readiness and execution. Importantly, the intervention improved knowledge and self-reported confidence, but its effect on actual prescribing behavior, antimicrobial utilization, and patient-level outcomes was not measured; reported intent to change practice should therefore not be interpreted as evidence of changed clinical behavior.

Overall, these findings reinforce that education is a necessary but insufficient strategy for sustainable antimicrobial stewardship. Rural-adapted ASP models incorporating ongoing education and external partnerships are needed to translate knowledge gains into consistent practice. While limited by low numbers of participants and lack of patient-level outcomes, the inclusion of participants from 38 rural hospitals enhances the relevance of these findings for similar resource-limited settings. Additional limitations should be noted. Survey responses were collected anonymously and could not be linked across time points, so paired comparisons at the individual level were not possible. Likewise, substantial attrition occurred across timepoints (30 pre-conference, 19 post-conference, and 11 at 6-month follow-up), and participants who completed later surveys may differ systematically from those who did not which may have inflated the apparent knowledge and confidence gains, inadvertently limiting validity and generalizability of findings. This study also lacked a control or comparison group, relied on self-reported knowledge and confidence rather than objective or behavioral measures, and used a self-developed survey instrument that was pre-tested but not independently validated.

## 4. Materials and Methods

This study employed a quantitative, quasi-experimental repeated cross-sectional design to evaluate the impact of the Georgia FLEX Antibiotic Stewardship Conferences held in 2023 and 2024. Sessions included didactic lectures, interactive workshops, and case-based learning focused on stewardship strategies facilitated by Infectious Disease (ID) Physicians, Pharmacists and Infection Prevention and Control (IPC) Practitioners. A similar curriculum, covering mechanisms of resistance, antibiogram use and interpretation, stewardship principles, diagnosis-based prescribing, and behavioral strategies for system-based change, was delivered across the 2023 and 2024 conferences, each consisting of a single full-day session. The evaluation was quantitative in design; no formal qualitative analysis was performed. Participants had roles in infection control or antimicrobial stewardship programs and included pharmacists, nurses, nurse practitioners, and other healthcare workers from 38 hospitals across Georgia.

In 2023, participants completed a pre-conference survey, a post-conference survey, and a 6-month follow-up survey using similar prompts to assess knowledge retention. Surveys were administered to the same cohort of conference attendees at each timepoint; however, responses were collected anonymously with no participant identifiers, so individual responses could not be linked across timepoints, and all statistical comparisons were carried out as unpaired analysis. In both 2023 and 2024, participants also completed a post-conference implementation survey, which used similar but distinct prompts from the initial 2023 conference surveys.

Survey questions assessed knowledge of antibiotic use, confidence in interpreting antibiograms, intent to change practice, and perceived barriers to ASP implementation. Survey instruments were developed by the study team for this educational program and were not previously validated. The survey instruments are provided as Supplementary Material. Quantitative survey responses were analyzed using descriptive statistics. Since all outcomes were categorical, we compared response distributions across the independent pre-conference, post-conference, and follow-up samples using chi-square tests, and used Fisher’s exact test when expected cell counts fell below 5. This happened often given the small sample and because responses tended to cluster in one or two categories. Significance was set at *p* < 0.05. Consent was obtained from all participants at the point of filling the surveys online, all data were anonymized, and ethical review was waived.

## 5. Conclusions

Regularly reinforced, targeted, low-cost education can serve as an effective implementation strategy to enhance antimicrobial stewardship in rural critical access hospitals. Addressing persistent structural barriers through system-level interventions such as protected time for ASP activities will be essential to translate educational gains into sustained stewardship practice and meaningful reductions in antimicrobial resistance.

## Figures and Tables

**Table 1:** Proportion of participants’ responses to planned implementation changes and primary barriers to antimicrobial stewardship implementation across the 2023 and 2024 conferences.

	2024 (*n* = 40)	2023 (*n* = 25)
**What type of changes do you plan to implement?**		
Apply the latest best practices	75%	76%
Utilize effective communication	25%	56%
Implement multidisciplinary approaches to work practice	50%	52%
Modify approaches for addressing adherence/compliance challenges	45%	44%
Modify approaches to supporting informational, emotional, and psychosocial needs of the healthcare provider	25%	16%
Change non-pharmaceutical approaches	10%	12%
Other	0%	0%
**Which of the following do you anticipate will be the primary barrier to implementing these changes?**		
Time constraints	56%	48%
Technology and digital limitations	27.5%	40%
Lack of tools and/or resources to support change	27.5%	16%
Cost	27.5%	12%
System constraints	20%	24%
Lack of multidisciplinary support	15%	20%
Lack of standardized best practices	10%	12%
Insurance/financial issues	0%	0%
Other	0%	4%

Chi-square comparisons between 2023 and 2024 responses were not statistically significant (all *p* > 0.05).

## Data Availability

The data presented in this study are available on reasonable request from the corresponding author.

## Supplementary Materials

The following supporting information can be downloaded at https://www.mdpi.com/article/10.3390/antibiotics15090859/s1: S1: Antimicrobial Stewardship Knowledge and Practice Survey; S2: Georgia Flex Antibiotic Stewardship Bootcamp Implementation and Evaluation Survey.
