# Supplementary material for: Educational Intervention Improves Antimicrobial Stewardship Knowledge but Reveals Persistent Barriers in Rural Hospitals
Source: Antibiotics (Basel). 2026 Sep 3;15(9):859. doi: 10.3390/antibiotics15090859 (PMC13603978; doi:10.3390/antibiotics15090859)
Supplement: Supplementary file 1 [file antibiotics-15-00859-s001.zip › antibiotics-4382920-supplementary.pdf]

S1_Antimicrobial Stewardship Knowledge and Practice Survey

Georgia Flex Antibiotic Stewardship Bootcamp
Georgia Department of Public Health

Purpose. This survey assesses knowledge, confidence, and practices related to antibiogram use and antimicrobial stewardship among healthcare professionals in rural and critical access settings. Your responses help evaluate and improve stewardship education.

Instructions. Please mark the box next to your response. Unless a question states "select all that apply," choose only one option. There are no right or wrong answers for opinion items; please answer honestly.

Confidentiality. Responses are collected anonymously and reported only in aggregate. Do not write your name or any identifying information.

Consent. Completion and submission of this survey will be taken as consent to participate in this exercise.

Q1. What is your core profession?

- ☐ Pharmacist
- ☐ Nurse
- ☐ Nurse Practitioner
- ☐ Doctor / Midwife / Physician's Assistant / Respiratory Therapist
- ☐ Other (please specify): ______________________

Q2. What is your predominant role?

- ☐ Infection specialist
- ☐ Non-infection specialist
- ☐ Generalist
- ☐ Management
- ☐ Scientist
- ☐ Other (please specify): ______________________

Q3. How many years have you been practicing in your current profession?

Years: ____

Q4. My hospital has an antibiogram.

- ☐ Yes
- ☐ No
- ☐ Unsure

Q8. In your current role, do your responsibilities involve contributing to your facility's antibiotic stewardship or infection control program?

- ☐ Yes
- ☐ Not directly
- ☐ No

Section B. Antibiogram Knowledge and Confidence

Q5. An antibiogram should be used to guide what type of therapy?

- ☐ Empiric
- ☐ Definitive
- ☐ Both empiric and definitive
- ☐ Unsure

Q6. "I feel confident reading and interpreting an antibiogram."

- ☐ Strongly agree
- ☐ Agree
- ☐ Neither agree nor disagree
- ☐ Disagree
- ☐ Strongly disagree

Q7. I would recommend antibiotics to treat the following conditions. *(select all that apply)*

- ☐ Gonorrhea
- ☐ Any middle ear infection (otitis media)
- ☐ Malaria
- ☐ Whooping cough
- ☐ Any acute pharyngitis / sore throat
- ☐ Any sinus infection
- ☐ Mpox
- ☐ Chicken pox or shingles
- ☐ Common cold or flu

Section C. Clinical Scenario

The following questions refer to a patient being considered for ciprofloxacin therapy for a suspected urinary tract infection (UTI).

Q9. Consulting the local antibiogram would be helpful to determine if ciprofloxacin is appropriate for this patient.

☐ Yes

☐ No

☐ Unsure

Q10. Would knowing the most common cause of UTI in the hospital or community help in determining if ciprofloxacin is the most appropriate antibiotic?

☐ Yes

☐ No

☐ Unsure

Q11. Would assessing cumulative local susceptibility rates of common UTI bacteria to ciprofloxacin be a good idea in this case?

☐ Yes

☐ No

☐ Unsure

Q12. If E. coli is the most common cause of UTI in the facility and the local resistance rate of ciprofloxacin to E. coli is only 18%, is that sufficient criteria to recommend ciprofloxacin as empiric therapy in this case?

☐ Yes

☐ No

Q13. Do you readily have access to your local resistance rates to E. coli or other bacteria?

☐ Yes

☐ No

☐ I don't know

Q14. Assuming that at the end of collecting UTI samples for 1 year there were 19 E. coli isolates in total, is this sufficient to construct your antibiogram?

☐ Yes

☐ No

☐ I don't know

Section D. Antimicrobial Stewardship

Q15. What estimated percent of antibiotics prescribed in the inpatient setting are unnecessary?

☐ 5%

☐ 10%

☐ 20%

☐ 30%

Thank you for completing this survey.

S2_Georgia Flex Antibiotic Stewardship Bootcamp Implementation and Evaluation Survey

Powered by Georgia Hospital Association

Purpose. This survey evaluates the Georgia Flex Antibiotic Stewardship Bootcamp educational activity and its impact. Your feedback guides program improvement and stewardship planning.

Instructions. For matrix items, mark one box per row. For other questions, mark all boxes that apply unless the item states otherwise. Please answer honestly; responses are collected anonymously and reported only in aggregate.

Section A. Evaluation of the Educational Activity

Q1. This activity supported the achievement of the core competencies listed below. *(mark one per row)*

Statement	Strongly agree	Agree	Neutral
**1.** Medical Knowledge (ACGME/ABMS)	☐	☐	☐
**2.** System-based practice (ACGME/ABMS)	☐	☐	☐
**3.** Apply Quality Improvement (IOM)	☐	☐	☐
**4.** Work in interdisciplinary teams (IOM)	☐	☐	☐

Q2. Please indicate the degree to which you agree that the activity achieved the following. *(mark one per row)*

Statement	Strongly agree	Agree	Neutral
**1.** The content was based on sound and current literature and/or research and/or best practices.	☐	☐	☐
**2.** The educational activity provided the opportunity to learn from and with other participants.	☐	☐	☐
**3.** The educational format was appropriate for the subject matter.	☐	☐	☐
**4.** The education, tools (if applicable), and resources in this activity provided useful information for my professional development.	☐	☐	☐
**5.** The activity improved my current knowledge and/or competence.	☐	☐	☐
**6.** The activity will help me improve my leadership skills, behaviors, and practices.	☐	☐	☐
**7.** The activity will help me engage my teams, internal and external customers.	☐	☐	☐
**8.** The activity will enhance my role within the interprofessional healthcare team.	☐	☐	☐

Section B. Intended Changes to Practice

Q3. Do you intend to change your work practice behavior?

☐ I do plan to implement changes in my work practice based on the information presented

☐ My current work practice has been reinforced by the information presented

☐ I need more information before I will change my work practice

Q4. What type of changes do you plan to implement? *(select all that apply)*

☐ Apply latest best practices

☐ Change non-pharmaceutical approaches

☐ Implement multidisciplinary approaches to my work practice

☐ Utilize effective communication

☐ Modify approaches for addressing adherence/compliance challenges

☐ Modify approaches to supporting the informational, emotional, and psychosocial needs of the healthcare provider

☐ Other (please specify): ______________________

Q5. How confident are you that you will be able to make your intended changes?

☐ Very confident

☐ Somewhat confident

☐ Not very confident

Q6. Which of the following do you anticipate will be the primary barrier to implementing these changes? *(select all that apply)*

☐ Lack of standardized best practices

☐ Cost

☐ Lack of tools and/or resources to support change

☐ Technology and digital limitations

☐ Time constraints

☐ System constraints

☐ Insurance/financial issues

☐ Lack of multidisciplinary support

☐ Other (please specify): ______________________

Section C. Program Information

Q7. How did you learn about this CME/CE activity? *(select all that apply)*

☐ Past participant

☐ Website

☐ Brochure

☐ E-mail / E-blast

☐ Colleague

☐ Other (please specify): ______________________

Thank you for completing this evaluation.
